# Do Coastal Areas Experience More Recession during the Economic Crisis—Evidence from China

**DOI:** 10.3390/ijerph191811361

**Published:** 2022-09-09

**Authors:** Juntao Tan, Xiaohui Hu, Fangdao Qiu, Hongbo Zhao

**Affiliations:** 1 School of Geography, Geomatics and Planning & Urban-Rural Integration Development Research Institute, Jiangsu Normal University, Xuzhou 221116, China; 2 School of Geography, Nanjing Normal University, Nanjing 210098, China; 3 School of Urban Resources & Environment, Jiangsu Second Normal University, Nanjing 210013, China; 4 Key Research Institute of Yellow River Civilization and Sustainable Development, Henan University, Kaifeng 475001, China

**Keywords:** resilience, regional economic resilience, coastal areas, Great Financial Crisis, China

## Abstract

The notion of resilience has been increasingly adopted in economic geography, concerning how regions resist and recover from all kinds of shocks. Most of the literature on the resilience of coastal areas focuses on biophysical stressors, such as climate change and some environmental factors. In this research, we analyze the regional economic resilience characteristics responding to the Great Financial Crisis in 2008 and its main determinants. We conclude that the coastal areas encountered more recession (or less growth) in the long term, and the secondary industry showed higher resilience than the tertiary industry. The influential factors of regional economic resilience varied across different stages of the crisis, and for the long term, good financial arrangement and governance ability could prompt the regional resilience to the crisis. Finally, some policy implications are proposed which may benefit dealings with major shocks such as economic crises and COVID-19.

## 1. Introduction

After the global financial crisis in 2008, global economic growth slowed, and the international situation was complicated and severe. This had a great impact on China’s economic development. Especially after the outbreak of COVID-19, the internal and external uncertainty of China’s economic development increased sharply, and the economic downturn pressure was huge. How to cope with downward pressure on the economy, prevent and resolve major risks, ensure that the economy is operating in a reasonable range, and build a resilient and healthy economic system has become the key to China’s economic development.

Resilience is a wide notion, which is often used to analyze the resistance to and recovery from shocks of systems. The notion is established in disciplines like physics and ecology, and is popular in economics, regional and social sciences, as well as in policy makers [1]. After the Great Financial Crisis in 2008, many scholars, especially economic geographers, have strengthened their interest in regional economic resilience by tackling the question of why some regions renew themselves and recover quickly, whereas others do not [2,3]. It has generated a boom in studies on regional economic resilience, including burgeoning conceptual debates and empirical research [4,5]. These studies attempt to clarify the heterogeneity of regional economic resilience in different regions, identify the underlying factors behind these differences, and make clear whether it is possible to influence these factors. The existing knowledge on this topic preliminarily derives from regions with developed economies (mainly Europe and North America) and is concerned about the national scale or larger. Few studies have focused on whether coastal areas with higher economic exposure encountered more recession and had low levels of adaptability. Coastal areas in China are embedded in a more open economy and government-oriented institutional contexts, and thus may have different economic resilience and determinants compared with regions in Europe and America. In short, the research on coastal areas in China can give some insights into whether economic resilience in China differs from some developed economies and whether higher economic exposure may cause lower resilience.

In this article, we put forward two main arguments. First, coastal areas with high exposure should have depressed more and had low economic resilience after the Great Financial Crisis, with different industries and different locations showing different resilience characteristics. Second, some positive factors such as adequate financial support, effective government policies, high-quality labor resource, and reasonable industrial structure can benefit resilience. Therefore, based on the conceptual framework of regional economic resilience, we quantitatively analyzed the economic resilience of coastal areas in China during the global financial crisis from 2008 to 2017, and then identified its main determinants using a panel regression model. Finally, some policy recommendations were proposed, which can bring some inspiration to deal with economic crises. The remainder of the paper is organized as follows: first, we reviewed the literature on regional economic resilience and its determinants in Section 2; then, we listed the main methodology and data in Section 3; thirdly, in Section 4, the main empirical results, including the characteristics of regional economic resilience and its determinants, were analyzed; and finally, in Section 5 and Section 6, the key findings and policy implications were discussed.

## 2. Literature Review and Theoretical Basis

### 2.1. The Concept of Resilience

Resilience usually refers to the ability of a social–economic system to recover from shocks, which may be economic crises, pandemics, natural disasters, etc. [6,7]. The concept of resilience has been popularized in the last decade, and it has been used in many different disciplines [8]. The nature of resilience is general conceptualized in three ways in the literature, namely engineering resilience, ecological resilience, and adaptive (evolutionary) resilience. Engineering resilience emphasizes a system’s ability to bounce back to a pre-recessional equilibrium state, and ecological resilience is defined as the scale of shocks that the system can absorb before its pre-recessional equilibrium state collapses [9,10]. These two notions adopt an equilibrium-based approach in the short term, which is usually criticized by economic geographers, and they have advocated an evolutionary approach to define resilience as a path-dependent process of creative destruction and constant renewal, as well as an open-ended reorientation, recoverability, and reorganization [11,12]. Just as Hassink proposed that resilience is more than a metaphor but less than a theory [13], it can be best described as a conceptual framework, and some useful conceptual frameworks have been put forward. In this article, we adopted the adaptive resilience and its related conceptual framework.

### 2.2. The Measurement of Regional Economic Resilience

Based on the notions of resilience, a large empirical body of literature has been published on the resilience, especially on regional economic resilience, after the Great Financial Crisis in 2008. The regional economic resilience is defined as the process of the regional economic system responding to the crisis and the ability to deal with the crisis. Since resilience is a highly complex and multi-dimensional concept, the measurement or assessment of regional economic resilience is difficult and challenging, and the methods may varied across different disciplines with different parameters [14,15]. The quantitative studies on regional economic resilience focused on different regional scales (e.g., European and national) with different parameters (e.g., GDP, unemployment). In the European context, Giannakis and Bruggeman assessed the economic resilience of Europe based on the employment changes from 2008 to 2013, and found that economic resilience showed a strongly uneven geography both in national patterns and within countries. The results indicated that regions in southern Europe were non-resilient, while the continental southern periphery was resilient [16]. Crescenzi et al. explored the short-term economic performance of regional resistance and found that the regional economic resilience, measuring with GDP and with unemployment rates, was quite different. The results showed that the Polish regions recorded the most positive economic resilience with GDP indicators, which was quite different to the results measuring unemployment [17]. In the national context, economic resilience of the U.K., the U.S., Italy, Australia, and some other development countries were measured with different methods, including the shift-share method, dynamic spatial panel, etc. [8,18,19,20]. Faggian et al. explored the regional economic resilience in terms of the local labor systems of Italy, and the results clearly pointed out high heterogeneous resilience [8]. Han et al. analyzed the economic resilience of U.S. counties with monthly employment data, and found each county exhibits unique action and reaction patterns for its recession and recovery processes, which was an important starting point for policy makers [19]. In short, the existing research on regional economic resilience is mainly derived from developed economies, particularly in Europe and the U.S., whereas regional economic resilience knowledge in China is inadequate.

In China, the regional economic resilience research is in its infancy, and the empirical research mainly focuses on some problem areas, such as resource-based cities and old industrial cities. Hu and Hassink provided a novel conceptual framework to better understand the long-term uneven resilience by exploring the notions of adaptability and adaptation, and then used it to explain the uneven resilience between Zaozhuang and Fuxin, two resource-based cities in China [21]. Thereafter, Hu and Yang, drawing on the concepts of institutional change and path development, analyzed the divergent economic resilience of two resource-depleted cities in China from an institutional change approach [22]. From a macro level, Tan et al. assessed the regional economic resilience in terms of resistance and recoverability and its influential factors of resource-based cities during economic crises in Northeast China and the whole of China, respectively [23,24]. In short, the quantitative studies of regional economic resilience at different scales and different regions all showed high heterogeneity, however, the argument about the difference in economic resilience between coastal and inland areas was insufficient.

Coastal areas concentrate a high proportion of human populations and economic activities, and these areas are also exposed to many hazards and risks, such as hurricanes, floods, and other disease epidemics [25]. Increasing risks along with highly degraded coastal ecosystems has sparked great work on the adaptation and social–ecological resilience of these areas [26]. Numerous stressors or disturbances have been identified that have influenced the ecosystems and human communities in coastal areas, and a recent bibliometric analysis showed that biophysical stressors, especial climate change (e.g., sea level rise) and some environmental factors (such as hurricanes, coastal erosion) are the main factors, however, the social–economic stressors only account for 25% [26]. The empirical research mainly focused on the resilience of coastal areas responding to some nature hazards, and proposed some planning strategies [27,28], but few paid attention to the economic crisis. The spatio-temporal evolutionary characteristics of marine economy resilience in three coastal areas of China were analyzed based on multi-dimensional perspectives [29]. The research on emerging small island developing states’ economies found that the interruptions in coastal cities were serious and affect both coastal cities and also their hinterlands, resulting in their individual and collective inability to produce and service enough food for local consumption and distribution to various catchment cities, so the resilience was low and needed sustainable development [30]. Since the economic linkages and the globalization of trade ties the regions more closely than before, the resilience of the coastal social–economic system is more tightly linked to larger-scale processes and exposed more to the global crisis, which may lead to more economic recession in coastal areas [25]. Therefore, identifying the characteristics of economic resilience to economic crises in coastal areas has become an urgent issue.

### 2.3. The Determinants of Regional Economic Resilience

Since the high heterogeneity of regional economic resilience, an important question arises as to why economic resilience might vary from region to region, and what factors determine the ability of regional economic resilience. In fact, the regional economic resilience is determined by a complex array of factors, such as labor conditions, industrial structures, technological coherence, financial arrangements, policies, government management, etc. [16,31,32]. Martin and Sunley proposed a framework of resilience determinants from four main subsystems, including the structural and business subsystem, the labor market subsystem, the financial subsystem and the governance subsystem [2]. The role of geography has been a key research motivation for why some factors have more impact on resilience in some regions, and not in other regions. Economic structure is an important determinant and even the most important one. Christopherson et al. found that a diversified economic base enabled a region to adjust and adapt [5]. Brown and Greenbaum tested the effect of industrial diversification in the Ohio counties of the U.S.A and found that counties with higher industrial diversification had higher resilience to external shocks [33], while Navarro-Espigares et al. provided evidence that service-intensive regions in Spain showed more resilience [34]. Considering the impact of labor conditions, human capital, and in particular education, is a major determinant for shaping regional economic resilience in Europe [16]. Bristow and Healy found that innovation leaders in Europe were significantly more likely to resist the crisis and recover quickly [35]. Kakderi and Tasopoulou found that national supporting policies explained not only resilience to the crisis, but also its vulnerability to the still ongoing crisis [36]. Furthermore, the quality of government was also an important factor shaping the regional resilience to the crisis [32]. In general, regional economic resilience is determined by multiple factors and those factors may vary across different regions, furthermore, the research mainly focused on the developed economies. Therefore, the effect of these factors and its mechanisms can be adapted to China is a topic worthy of further investigation.

Through this literature review, we found that regional economic resilience at different regions shows a heterogeneous pattern and is determined by multiple factors. However, the research of regional economic resilience in coastal areas and empirical research in China is insufficient. Based on this literature review, we made two theoretical hypotheses. First, coastal areas which were exposed more to the global crisis may have encountered more recession or less growth in the economic crisis compared with other regions. Second, considering the institutional differentiation between China and Western countries, we argued that the effect of some factors may be different.

### 2.4. Conceptual Framework

**Figure 1:**
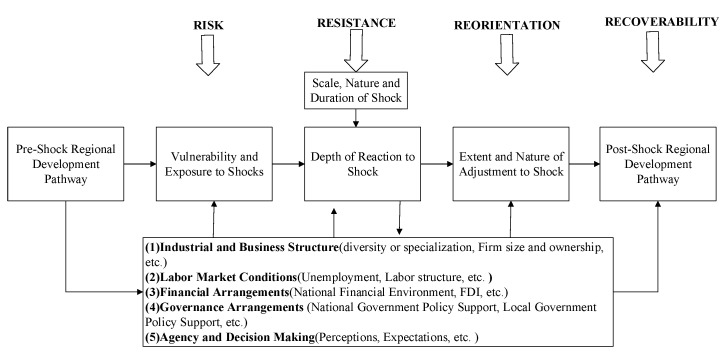
The conceptual framework of regional economic resilience. Source: Adapted from Martin et al. (2016) [2].

Some useful frameworks for analyzing regional economic resilience have been put forward. Martin provides a useful conceptual framework, that regional economic resilience includes four dimensions or aspects to recession (or shock), namely resistance, recovery, reorientation, and renewal [20]. Martin et al. revise and develop the framework and emphasize that resilience is a multifaceted process with several phases [2]. Regional economic resilience can be viewed as comprising the following four sequential steps: the risk (or vulnerability) of the region to the shock; the resistance to the shock; the ability to undergo the adjustments and adaptations necessary to resume the region’s core functions, which we can call (adaptive) reorientation; and finally, the degree of recoverability from the shock (Figure 1).

These sequential aspects of the regional economic resilience process depend on the nature, duration, and depth of the shock, on the prior growth path, and on the various determinants of that growth path. Based on previous studies, these determinants of the regional economic resilience can be generally divided into five aspects (Figure 1) [16,31,32]. First, industrial and business structures influence regional economic resilience. Second, labor market conditions, including the skill profile of the labor force, gender profile, and labor structure, affect regional economic resilience. Third, the financial arrangement, including the national financial, FDI, and other types of financial support, can affect the resilience. Fourth, the government arrangements (national and local) and finally the agency, decision making, and experiences dealing with past shocks can also affect the regional economic resilience [32].

## 3. Materials and Methods

### 3.1. Study Area and Data

**Figure 2:**
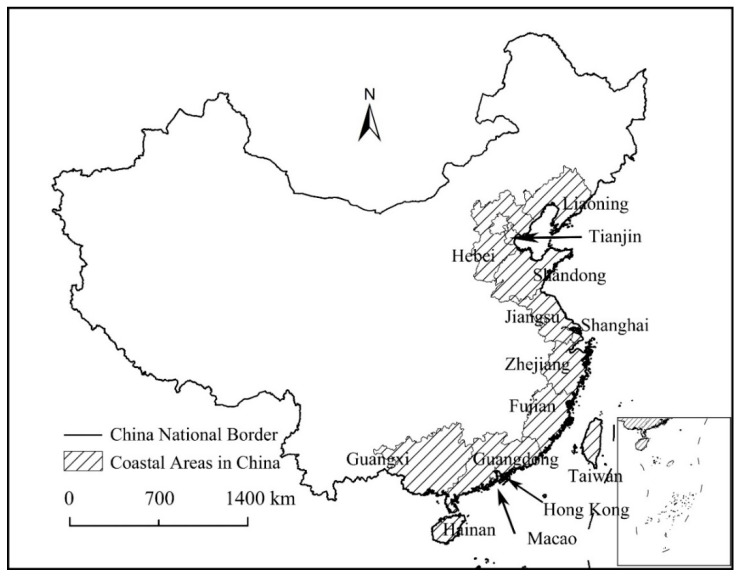
The location of Coastal Areas in China (authors’ own composition).

Coastal areas in China officially include Liaoning, Shandong, Hebei, Jiangsu, Fujian, Zhejiang, Guangdong, Fujian, Hainan, and Taiwan, totaling 10 provinces, and Tianjin, Shanghai, Macao, and Hong Kong, totaling 4 cities. Given the data availability, Taiwan, Hainan, Macao, and Hong Kong are not included in this research. Therefore, the study area of coastal areas in this paper included eight provinces and two municipality, totaling 112 cities (Figure 2). In 2008, the total GDP of the study area was 17,111.9 billion CNY (China Yuan), accounting for 63.35% of the national total. In 2017, the total GDP of the study area was 49,458.7 billion CNY (China Yuan), accounting for 59.44% of the national total. We could find that the total GDP of the study area accounted for about 60% of the national total from 2008 to 2017. In 2017, the average GDP per capita was 76,828 CNY, much higher than the national average (53,980 CNY), and the total value of imports and exports of goods was 21,721.3 billion CNY, accounting for 78.11% of the national total, and the total foreign investment accounted for 75.19% of the national total. We could find that coastal areas in China are the main areas of foreign trade and foreign investment, and the level of economic development was high.

The time scope of this study was from 2008 to 2017. The data was derived from China City Statistical Yearbook from 2008 to 2018, China Statistical Yearbook for Regional Economy from 2008 to 2014, and the Statistical Yearbook of each province and municipality from 2015 to 2018.

### 3.2. Measuring Regional Economic Resilience

In this study, we focus specifically on measuring two of the four dimensions of regional economic resilience showing in Figure 1, namely resistance and recoverability. Several different methods were used to measure economic resistance and recoverability. In this article, we use the method proposed by Martin to measure regional economic resilience using Gross Domestic Product (GDP) data [2]. Since resilience is concerned with how different regions are affected by a common recession, then the expected economic change in region r during a recessionary period would be given as:

(1) $${\left(\Delta E_{r}^{t+k}\right)}^{\mathrm{expect}}=\underset{i}{\sum }E_{\mathrm{ir}}^{t}{*g}_{N}^{t+k}$$

where represents the expected change of economic output (GDP) of region r in the contraction period (t + k), $E_{\mathrm{ir}}^{t}$ represents the economic output (Value Added) of industry i of region r at starting time t, in this research t was 2007, and $g_{N}^{t+k}$ represents the change rate of national GDP in t + k time, k was from 1 (year 2008) to 10 (year 2017) in this research. Thereafter, the regional resistance can be measured as:

(2) $${\mathrm{Resis}}_{r}=\frac{\left(\Delta E_{r}^{t+k}\right)-{\left(\Delta E_{r}^{t+k}\right)}^{\mathrm{expect}}}{\left|{\left(\Delta E_{r}^{t+k}\right)}^{\mathrm{expect}}\right|}$$

where $\Delta E_{r}^{t+k}$ represents the actual change of GDP of region r in the contraction period (t + k). The value of ${\mathrm{Resis}}_{r}$ was around zero, and when the value of ${\mathrm{Resis}}_{r}$ was positive, a region was more resistant to the shock than the national average level. This study focuses on the Great Financial Crisis that occurred in 2008. The GDP growth rate of coastal areas experienced roughly the same development trend as China, which peaked in 2007, declined in 2008, recovered briefly in 2010, and then declined again. After 2017, China’s economy has begun to be impacted by new disturbances such as Sino–U.S. trade friction and COVID-19. We define the period from 2008 to 2017 as the recession period and use the economic resistance to indicate the regional economic resilience.

### 3.3. Measuring the Difference of Regional Economic Resilience

To measure the development trend of regional economic toughness and regional differences in different years, we calculated the mean value and standard deviation of regional economic resilience as follows:

(3) $$Mean=\sum {\mathrm{Resis}}_{r}/n$$

(4) Deviation=∑(Resisr2−(∑Resisr)2n)n−1

where ${\mathrm{Resis}}_{r}$ is the value of economic resilience in region r; *n* is the number of research units, totaling 112 cities. In our research all regions are within the calculation range and there are no outliers.

### 3.4. Detecting Influential Factors

Based on the conceptual framework in Figure 1, we selected ten indicators from five aspects to detect the potential factors influencing the economic resilience of coastal areas in China. We selected per capita GDP (CNY) (*PCGDP*) and foreign trade dependence (ratio of total value of imports and exports of goods to GDP, %) (*FTD*) to present the regional development level. Thereafter, we selected industrial diversity (*DIV*) and the proportion of gross industrial output value above designated size in GDP (%) (*GOV-ADS*) to reflect the industrial structure. Industrial diversity was calculated by using an entropy index based on the regional sector employment data as follows.

(5) DIV=∑i=1n(erier)∗ln(1erier)=−∑i=1n(erier)∗ln(erier) 

where *DIV* is the industrial diversification in region *r*, and n is the number of total industries; $e_{ri}\;$ is the employment of industry *i* in region *r* and, and $e_{r}$ is the total employment in region *r*; ln means the natural logarithm. We selected the proportion of employees in the tertiary industry (Employees in the tertiary industry/Number of employed persons, %) (*Tertiary*), proportion of employees in manufacturing (Employees in the manufacturing/Number of employed persons, %) (*Manufacture*), and registered unemployed rate in urban areas (%) (*Unemployed*). These three factors can represent the labor conditions from employment rate and labor structure, and they can also reflect the industrial structure to a certain extent. The ratio of local fiscal revenue to GDP (%) (*Govern-Fiscal*) and ratio of foreign direct investment to GDP (%) (*FDI*) were selected to present the financial arrangement, and the ratio of fixed asset investment to GDP (%) (*Fix-Invest*) was selected to express the ability of governance. To examine the potential factors influencing regional economic resilience, referencing previous research [37] we estimated a panel regression model as follows:

(6) $$Y_{it}=\alpha +\beta_{1}PCGDP_{it}+\beta_{2}FTD_{\mathrm{it}}+\beta_{3}DIV_{\mathrm{it}}+\beta_{4}GOV\_ADS_{it}+\beta_{5}{\mathrm{Tertiary}}_{it}+\beta_{6}Manufacture_{it}+\beta_{7}Unemployed_{it}\;+\beta_{8}Govern\_Fiscal_{it}+\beta_{9}FDI_{it}+\beta_{10}Fix\_Invest_{it}+u_{it}.$$

where $y_{it}$ is the dependent variable (regional economic resilience in terms of resistance in region i of t year). $PCGDP_{it},FTD_{it}\ldots \ldots Fix\_Invest_{it}$ are ten independent variables. $\beta_{1}\ldots \ldots \beta_{10}$ are coefficients, α is coefficients, and $u_{it}$ the error, varies over region *i* and time *t*. The error $u_{it}$ is used to decide to select fixed effects or random effects model. In a fixed effects model, $u_{it}$ is assumed to vary non-stochastically over *i* or *t* making the fixed effects model analogous to a dummy variable model in one dimension. In a random effects model, $u_{it}$ is assumed to vary stochastically over *i* or *t* requiring special treatment of the error variance matrix [38].

## 4. Results

### 4.1. Economic Resilience of Coastal Areas in China

**Figure 3:**
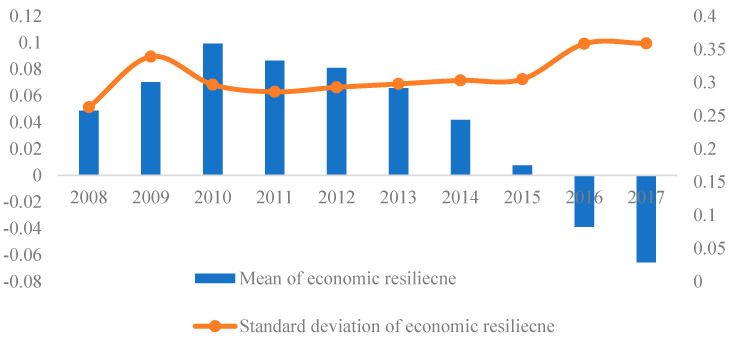
The mean and standard deviation of economic resilience of coastal areas in China.

Calculating the mean and standard deviation of the economic resilience of 112 cities in China’s coastal areas, shown in Figure 3, we could find two interesting results. First, the mean value of economic resilience of coastal areas was not always low as we expected; only after 2015 the mean value becoming negative. Second, the economic resilience of coastal areas shows different laws in different stages of the crisis, and the mean value of regional economic resilience presents a trend of rising first and then continuing to decline.

When the economic crisis broke out in 2008, the economic resilience of coastal areas was slightly higher than the national average. Although the economy of coastal area was greatly affected by the crisis, the regional economic development foundation was good. Therefore, the regional economic resilience was still higher than the national average. After 2008, the economic resilience of coastal areas continued to increase and reached a peak in 2010. The main reason for this development trend was the implementation of the four trillion investment plan (four trillion RMB investment). In order to ensure the stable development of the national economy and to reduce the impact of the economic crisis on China, the State Council issued an economic stimulus plan in the end of 2008, and cooperated with the loose monetary policy. These macro-regulations had a short-term stimulating effect on the growth of the economy of coastal areas in China, and promoted the increase of regional economic resilience.

After 2010, the economic resilience of coastal areas continued to decline and was lower than the national average in 2016 and 2017. The continuous decline of economic resilience in coastal areas after 2010 can be explained from the following three aspects. First, the cessation of the four trillion investment plan stopped economic growth driven by government investment in the early stages of the economic crisis. Second, the global economy fell into a deep recession due to the economic crisis, which hit China’s foreign trade exports severely, and these exports were mainly distributed in the coastal areas of China. The foreign trade dependence decreased from 48.84% to 33.62% from 2010 to 2017. Finally, the Chinese economy has entered a “new normal” stage since 2014, which means the economy has shifted gear from the previous high speed to a medium-to-high speed growth. It was accompanied by the issuance of policies that support the supply-side reforms to optimizing the existing industry structure. Coastal areas are key areas for reform, whose economic resilience inevitably declines in short. The standard deviation of economic resilience of coastal areas shows a rising trend, indicating that the regional difference of economic resilience in coastal areas is slowly increasing. This is highly related to the further strengthening of the spatial agglomeration characteristics of regional economic resilience. For example, by 2017, the Bohai Rim region has become a low-value area of economic resilience, while the economic resilience of most cities in Zhejiang and Fujian are significantly higher than the national average, the difference of regional economic resilience increasing slowly.

**Figure 4:**
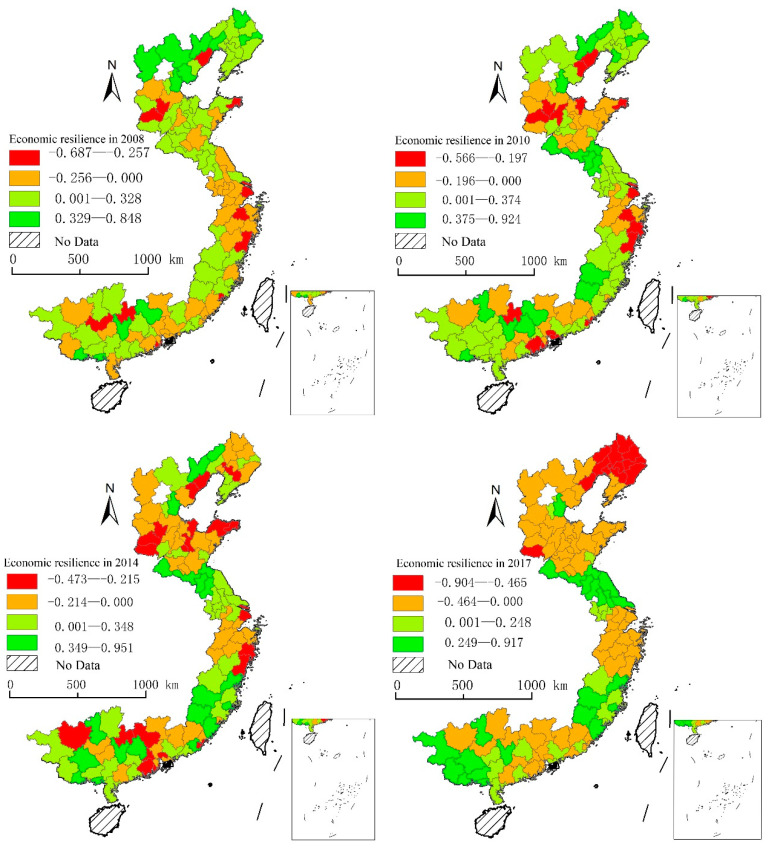
Economic resilience of coastal areas in China.

As we cannot present a table with the value of economic resilience of 112 cities of coastal areas in China from 2008 to 2017, we selected four time nodes in 2008, 2010, 2014, and 2017 based on the development trend of regional economic resilience. Thereafter, we analyzed the spatial distribution of regional economic resilience at different stages of the economic crisis (Figure 4). We classified the area using the natural breaks method of ArcGIS, whose class breaks are created in a way that best groups similar values together and maximizes the differences between classes. The 112 cities of coastal areas were divided into four categories based on the value of regional economic resilience, named low area, sub-low area, sub-high area, and high area. The economic resilience value of the low and sub-low areas was lower than the national average value, while the sub-high area and the high area were higher than the national average value.

In 2008, 48 cities belonged to the low area and the sub-low area, which mainly distributed near the Yangtze River Delta, Southern Hebei Province and some coastal cities, and the spatial agglomeration characteristics were not very significant. In 2010, 47 cities had lower economic resilience than national average, and the spatial agglomeration has increased significantly. It formed three low-value agglomeration areas; Shandong–Hebei Province agglomeration area, Southern Jiangsu–Shanghai agglomeration area, and Guangdong Province agglomeration area, respectively. In 2014, the number of low areas and sub-low areas increased to 60. When compared with 2010, except three previous low-value agglomeration areas, Southern-middle Liaoning Province become the new low-value agglomeration area. In 2017, the number of cities with lower economic resilience increased to 72, and the characteristics of spatial agglomeration became more obvious. The Bohai Rim area composing of Liaoning, Hebei, and Shandong become the biggest low-value agglomeration area, and the low area cities mainly distributed in Liaoning Province. This phenomenon was explained for two reasons. First, the economy of Northeast China encountered a cliff-like decline after 2014, and some cities had a negative economic growth, which as *The Economist* said, was “back in the cold.” In addition, it had a high relationship with the falsification of statistical data in Liaoning Province from 2011 to 2014, which made the fiscal revenue falsely increase by about 20%. In short, the number of cities with lower economic resilience in coastal areas increased and showed a trend of agglomeration.

### 4.2. Economic Resilience of Different Industries

**Figure 5:**
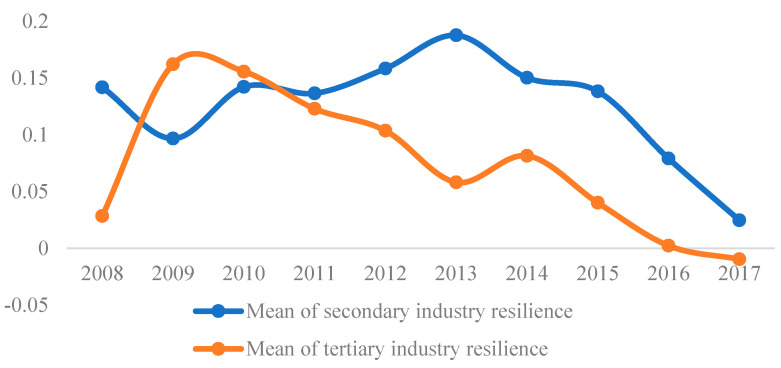
Mean of secondary and tertiary industries’ resilience of coastal areas in China.

The resilience of different industries in coastal areas during the economic crisis were analyzed in this section. Since the proportion of the primary industry was low, and it was mainly affected by climate rather than the economic crisis, we focused on the resilience of secondary and tertiary industries in this research. We calculated the resilience of the secondary and tertiary industries of 112 cities in coastal areas, and its mean of each year (Figure 5). We found three main results. First, the resilience of the secondary industry was generally higher than that of the tertiary industry, which indicated that the impact of the economic crisis on the secondary industry was lower than the tertiary industry. Second, the resilience of the tertiary industry presented an inverted U-shaped development trend that rose first and then declined. When the economic crisis broke out in 2008, the tertiary industry suffered a great recession, and the mean of resilience was close to zero. This was highly related to the fact that the economic crisis directly affected the financial markets. With the implementation of the four trillion investment plan, the resilience of the tertiary industry recovered rapidly in 2009 and 2010. After 2010, the resilience declined continually, which may have been related to the sluggish imports by Western countries. Finally, the resilience of the secondary industry showed an inverted N-shaped curve development trend that decreased first, then increased, and then decreased. The resilience of the secondary industry was high when the economic crisis broke out in 2008, and then declined in 2009. This illustrated that the impact of the economic crisis on the secondary industry was lagging, and also demonstrated that the industrial transmission mechanism of the crisis was to affect the tertiary industry first and then the secondary industry. The resilience of the secondary industry showed an upward trend from 2009 to 2013, indicating that the effect intensity and duration of the macro-stimulus on the secondary industry was significantly higher than that of the service industry. In short, the resilience of the secondary industry was higher than the tertiary industry, and the impact of the crisis on the secondary industry lagged behind the tertiary industry.

**Figure 6:**
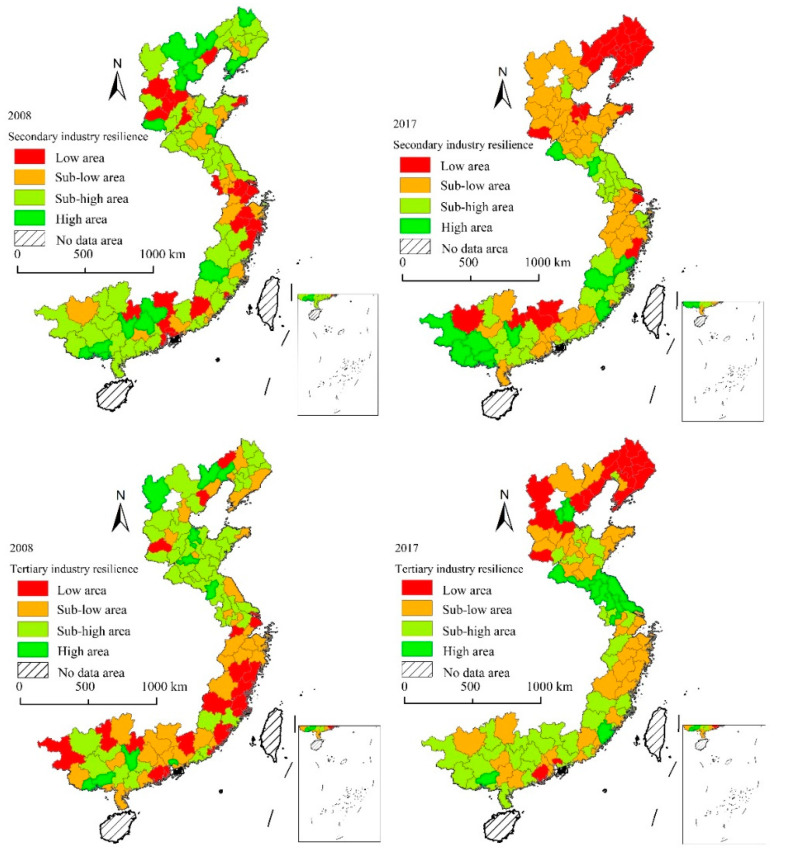
Resilience of secondary and tertiary industries coastal areas in China.

We analyzed the spatial distribution of secondary and tertiary industry resilience at different stages of the crisis, and we divided the cities into four categories (Figure 6). When the economic crisis broke out in 2008, there were 42 cities belonging to the low and sub-low areas of the secondary industry resilience, and 57 cities belonging to the low and sub-low areas of the tertiary industry resilience. Cities with low secondary industry resilience were mainly distributed in Hebei, Shanghai, Southern Jiangsu, and Northern Zhejiang, and the low value of tertiary industry resilience was mainly concentrated in the south of coastal areas including Shanghai, Zhejiang, Fujian, and Guangdong. In 2017, the number of cities with low secondary and tertiary resilience increased to 70 and 62, respectively. The low-value agglomeration areas of secondary and tertiary industry resilience had high coincidence, and were mainly distributed on the Bohai Rim area, Southern Jiangsu, and the Northern Zhejiang area. Both secondary and tertiary industry resilience in low area cities was mainly concentrated in Liaoning Province.

### 4.3. Economic Resilience of Different Location

**Figure 7:**
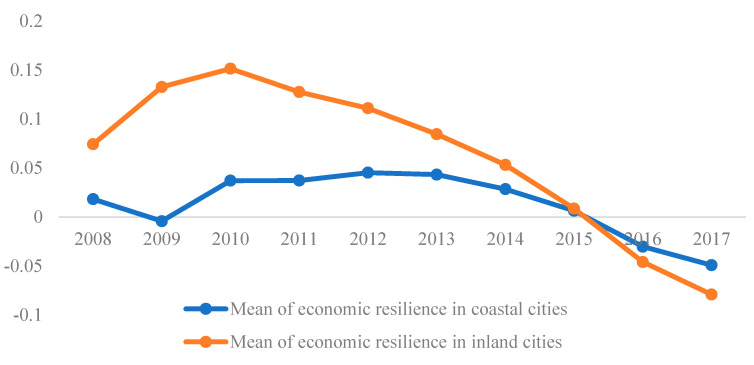
Mean of economic resilience of coastal cities and inland cities.

Based on the location condition, 112 cities were divided into two types, namely coastal cities and inland cities, and there are 51 coastal cities and 60 inland cities. We calculate the mean of economic resilience of two type cities (in Figure 7), and we can draw the following main results. First, the mean of economic resilience of inland cities was higher than that of coastal cities from 2008 to 2015. The main enabling factor can be identified from foreign trade. Coastal cities were the main carrier of China’s foreign trade and were inevitably directly affected by the crisis. This conclusion further validated the spatial transmission mechanism of the economic crisis that the effect of the economic crisis spread from coastal cities to inland cities. Second, the mean value of coastal cities began to surpass that of inland cities after 2015. This is mainly because with the impact of the economic crisis being weakened, the economic growth rate of coastal cities has begun to surpass that of inland cities due to their location advantages.

### 4.4. Influential Factors of Regional Economic Resilience

We applied a panel regression model to examine the determinants of regional economic resilience from 2008 to 2017, and the results were estimated by Stata version 14.0. The regional economic resilience had different features at different stages of the economic crisis, which showed an increasing trend from 2008 to 2010, a slow decline from 2011 to 2015, and a rapid decline after 2015, lower than the national average, therefore, we checked the influential factors of regional economic resilience from three short periods: 2008 to 2010, 2011 to 2014, and 2015 to 2017, and the results are shown in Table 1. To ensure the accuracy of the regression results, an F-test and a Hausman test were used to test the regression models. The F-test was used to check whether a fixed effect is better than OLS regression. The Hausman test was used to examine if a fixed effect is better than random effect. If the Hausman test was significant, we selected the fixed effects model, if not, we selected the random effects model.

In the long term, we may draw the following results. First, except labor conditions, urban development, industrial structures, financial arrangements, and governance, all had a significant effect on regional economic resilience, but the detail indicator and its effect direction were different. Second, two urban development indicators, *PCGDP* and *FTD*, had a negative effect on regional economic resilience at a 99% confidence level, and we can infer that regions with high GDP per capita and foreign trade dependence encountered more recession during this economic crisis. Third, two indicators of industrial structure had different effects on regional economic resilience. *DIV* had a negative effect, while *GOV-ADS* had a positive effect, which indicated that regions with a high proportion of large-sized enterprises and low industrial diversification were less affected by this crisis. Finally, indicators of financial arrangements and governance all had a positive effect on regional economic resilience, and we can conclude that good financial support and governance ability can improve the regional economic resilience.

From the regression results, we could find that the factors affecting regional economic resilience varied across different stages of the crisis. At the beginning of the crisis (2008–2010), *FTD* and *Tertiary* had a significant negative effect, while *DIV*, *Govern-Fiscal* and *Fix-Invest* had a significant positive effect. At the middle of the crisis (2011–2014), *DIV* had a significant negative effect, while *GOV-ADS*_,_ *Tertiary*, *Manufacture,* and *Fix-Invest* had a significant positive effect. In the post-crisis era (2015–2017), *Tertiary* had a significant negative effect, while *GOV-ADS*, *Govern-Fiscal*_,_ *FDI,* and *Fix-Invest* had a significant positive effect. Based on the effect of different influential factors at different stages, we divided the 10 influential factors into four categories, namely positive factors, negative factors, bidirectional factors, and insignificant factors.

*GOV-ADS*, *Manufacture*, *Govern-Fiscal,* and *FDI* were positive factors. Except the period from 2008 to 2010, *GOV-ADS* always had a significant positive effect on the resilience. Compared with large and medium-size enterprises, especially the large state-owned enterprises, small and medium-sized enterprises usually had problems such as small-scale development and a single production structure. Their ability to cope with economic crises was weak, and many enterprises even went bankrupt. Therefore, regions with a high proportion of output value of above designated size enterprises had higher resilience. *Manufacture* had a significant positive effect from 2011 to 2014, and it was consistent with previous research results that the secondary industry had higher resilience in this crisis. The effect of *Govern-Fiscal* and *FDI* was generally significant, and we can conclude that financial supporting from both government fiscal and FDI can improve the resilience.

*PCGDP* and *FTD* were negative factors. From a long period, these indicators had a significant negative effect, while *PCGDP* was not significant in the short period, and the *FTD* was significant at the beginning of the crisis. This result also validated that the economic crisis affected the foreign trade directly at the beginning of the crisis. *DIV*, *Tertiary,* and *Fix-Invest* were bidirectional factors. At the beginning of the crisis, industrial diversity had a significant positive effect, while *Tertiary* was negative, and they had an opposite effect in the middle of the crisis. At the beginning of the crisis, regions with a diversified industry and a low proportion of the tertiary industry showed higher resilience, and it verified the conclusion that the tertiary industry had low resilience at the beginning of the crisis. In the middle of the crisis, *Fix-Invest* had a negative effect on the resilience, while other times, it had a significant positive effect. Therefore, fixed asset investment can improve regional resilience most of the time, and it shows that high government management ability can increase the resilience. *Unemployed* was the only insignificant factor, and we can conclude that the unemployment data in China was not very suitable for the research of resilience. This was mainly because the registered unemployed persons in China only included the persons with non-agricultural households and who had been registered at the local employment service agencies to apply for a job. It did not include the persons with an agriculture household, and the unemployment rate was stable, as it was less affected by the financial crisis.

## 5. Discussion

### 5.1. Economic Resilience Characteristics of Coastal Areas

The results show that the economic resilience of coastal areas in China was not always as low as we expected in our first hypothesis, and the regional economy experienced a short-term recovery which was higher than the national average, but for the long term, coastal areas encountered a deeper recession (or less growth) than the national average. Since the evolutionary resilience concerning the long-term ability of regions to reconfigure their social–economic structure or to develop new growth paths [39,40], when conducting empirical research, how to choose the time scale of research becomes a controversial issue. In addition, our research identified that most of the time, the economic resilience of coastal cities is lower than that of inland cities. These findings are consistent with some previous research that the recent global economic crisis and its immediately preceding boom have had profound impacts on the world’s coasts and the coastal areas experienced a double exposure to the economic crisis and climate change [41,42]. Therefore, in future research, we should pay attention not only to natural disasters, which may be caused by climate change, but also to social–economic shocks (e.g., economic crises) in coastal areas. Previous research on regional economic resilience showed high spatial heterogeneity both in national patterns and within countries [16,43], and our research added some evidence of heterogeneity between coastal areas and inland areas.

### 5.2. Determinants of Economic Resilience of Coastal Areas

Our results suggest that the influential factors of regional economic resilience varied across different stages of the crisis. This result deepens the findings of previous research that the effect of the main factors may vary across different economic crises [24], and also verifies the view that the local response to the economic crisis varied according to the specific origins and characteristics of the crisis [31]. For the long term, regional development levels in terms of GDP per capita and foreign trade dependence all had negative effects, and this is consistent with previous research of resource-based cities in China that these cities with a low development level and a remote location had higher resilience [24]. However, this seems to be contrary to empirical research in Europe that regions with a higher development level before 2008 faced less severe impacts of the crisis [16], and this can explain from different research scales and research areas.

For the long term, the diversification of regional economies seems to negatively affect their resilience in our research, and this echoes previous studies on European cases that specialization seems to have a positive effect [16]. Just as Xiao et al. stressed, diversification gives regional economic growth a higher and stronger anti-risk ability [44]. In addition, the model suggests that industrial enterprises above the designated size seem to have had a positive effect. In comparison to large-scale enterprises, small- and medium-scale enterprises are highly vulnerable to hazards due to their limited risk-management approaches and less capital base [45]. We also found that good financial arrangements and governance ability can prompt the regional economic resilience to crisis. These findings are aligned with the previous research that both supporting policies and high quality of government are positive factors shaping regional economic resilience [32,36].

### 5.3. Policy Implications

The understanding of the nature of regional economic resilience and its determinants to the Great Financial Crisis in coastal areas of China can help to generate some policy implications for responding to the crisis. Firstly, more attention and policy support should be paid to the coastal areas to reduce the crisis exposure and increase the regional economic resilience, since these areas usually encounter more recession during the crisis in the long term. Second, although foreign trade dependence has a negative effect, more complex foreign trade systems should be established which means more diversified products, export destination, and product origin. This is because resilience is often cited as an important attribute of complex systems. At the same time, target policies should be proposed to stimulate consumption and expand domestic demand in coastal areas. Third, both central and local governments should accelerate the implementation of numbers of policies to promote the development of small and medium-sized enterprises, such as increasing financial support and reducing costs and burdens. Fourth, both immediate relief measures and long-term planning are needed to reduce recession and re-energize the economy in a crisis [46]. Finally, the Great Financial Crisis of 2008 is different in origin and nature from the crisis as a result of COVID-19, and our research also indicated the factors affecting regional economic resilience varied across different stages of the crisis. Therefore, when we formulate regional development policies for the post-epidemic era, the research conclusions of this article can be used as a reference basis, and the process and differences of the influence of COVID-19 on regions, industries, and enterprises should be considered more.

## 6. Conclusions

Based on the conceptual framework of regional economic resilience, this paper analyzed the economic resilience of coastal areas in China after the Great Financial Crisis in 2008, and then identified its main determinants using a panel regression model. We draw five key conclusions. First, although the economic resilience was not low at the beginning of the crisis as expected, and even experienced a higher short-term recovery, the coastal areas encountered a deeper recession (or less growth) than the national average in the long term, and the cities with lower economic resilience present clustering features. Second, the resilience of the secondary industry was higher than tertiary industry, and the impact of the crisis on the secondary industry lagged behind the tertiary industry. Third, within the coastal areas, the resilience of inland cities was generally higher than the coastal cities. Fourth, the influential factors of regional economic resilience varied across different stages of the crisis, and some factors even had the opposite effect at different stages. Finally, for long-term, regional development levels in terms of GDP per capita and foreign trade dependence, diversification of regional economies had a negative effect, while good financial arrangements and governance ability could prompt the regional resilience to a crisis.

Although some works have been done in this paper, still, there are several inadequacies and possible extensions for the future. First, in this paper the GDP data were selected to measure the economic resilience, and other types of data can be used, such as financial data and import and export data. Second, in this article, the method to measure the regional economic resilience is relative to the national average, and it is difficult to reflect endogenous regional resilience, therefore, better methods need be used to reflect the region’s real expectations of growth over time in the further research. Finally, when detecting the influential factors of regional economic resilience, we used a panel regression model which ignored the spatial effects. The spatial panel regression can be considered in further research which can deal with interaction effects among geographical units. The main advantage of working with spatial panels is that we can control for spatial and time specific effects, and we can use this model to test for the existence of spatial interaction effects, and related to that, spatial spillover effects [47]. Thereafter, we can detect the influences in regional economic resilience in more detail.

## Figures and Tables

**Table 1:** The Regression results of the main influential factors on regional economic resilience.

	2008–2017		2008–2010		2011–2014		2015–2017	
	Coef.	*p*-Value	Coef.	*p*-Value	Coef.	*p*-Value	Coef.	*p*-Value
*PCGDP*	−8.53 × 10^−7^ ***	0.004	−2.03 × 10^−6^	0.117	−1.16 × 10^−7^	0.490	1.89 × 10^−7^	0.854
(X_1_)	(2.96 × 10^−7^)		(1.30 × 10^−6^)		(1.69 × 10^−7^)		(1.03 × 10^−6^)	
*FTD*	−0.0105 ***	0.001	−0.0069 *	0.066	−0.0051	0.261	−0.0049	0.248
(X_2_)	(0.0030)		(0.0038)		(0.0045)		(0.0043)	
*DIV*	−0.1961 **	0.026	0.4388 **	0.016	−0.1689 **	0.018	0.3385	0.111
(X_3_)	(0.0799)		(0.1819)		(0.0709)		(0.2114)	
*GOV-ADS*	0.1619 ***	0.000	0.0581	0.297	0.0935 ***	0.000	0.2779 ***	0.000
(X_4_)	(0.0218)		(0.0557)		(0.0227)		(0.0354)	
*Tertiary*	−0.00013	0.945	−0.006 *	0.051	0.0039 ***	0.004	−0.0084 **	0.036
(X_5_)	(0.0018)		(0.0031)		(0.0014)		(0.0039)	
*Unemployed*	0.0068	0.211	−0.0141	0.159	−0.0037	0.404	0.0028	0.758
(X_6_)	(0.0054)		(0.0100)		(0.0044)		(0.0092)	
*Manufacture*	−0.00001	0.696	0.0020	0.558	0.0004 ***	0.000	−0.0038	0.407
(X_7_)	(0.00003)		(0.0034)		(0.00008)		(0.0046)	
*Govern-Fiscal*	0.0049 ***	0.000	0.01417 **	0.017	−0.0004	0.543	0.0065 **	0.012
(X_8_)	(0.0011)		(0.0059)		(0.0006)		(0.0025)	
*FDI*	0.1592 ***	0.000	0.0782	0.197	−0.0722	0.105	0.1434 ***	0.000
(X_9_)	(0.0295)		(0.0607)		(0.0444)		(0.0289)	
Fix-Invest	0.0014 ***	0.002	0.0022 *	0.053	−0.0016 ***	0.002	0.0012 **	0.045
(X_10_)	(0.0004)		(0.0011)		(0.0005)		(0.0006)	
Constant	−0.1815 **	0.012	−0.8719 **	0.060	0.1705	0.173	−0.9291 *	0.078
	(0.1388)		(0.4580)		(0.1245)		(0.5249)	
Number of obs	1119		336		447		336	
R-squared	0.1650		0.2462		0.1708		0.6090	
F-test	F(111,997) = 22.71 Prob > F = 0.0000		F(111,214) = 8.43 Prob > F = 0.0000		F(111,325) = 59.65 Prob > F = 0.0000		F(112,214) = 60.34 Prob > F = 0.0000	
Hausman test	47.03 ***		20.02 *		178.97 ***		60.07 ***	
Estimation Model	Fixed effects model		Random effect model		Fixed effects model		Fixed effects model	

*** *p* < 0.01, ** *p* < 0.05, * *p* < 0.1.

## Data Availability

Not applicable.
